# Unboxing disability communication: Exploring the methodological contours of critical disability research in public health archives

**DOI:** 10.1016/j.ssaho.2026.102668

**Published:** 2026-05-26

**Authors:** Aparna Raghu Menon, Tanya Titchkosky

**Affiliations:** a Dalla Lana School of Public Health, University of Toronto, Canada; b Department of Social Justice Education, Ontario Institute for Studies in Education of the University of Toronto, Canada

**Keywords:** Communication, Public health, Archives, Interpretive methodology, Disability studies

## Abstract

This article provides an account of the experience of conducting archival research on disability, including its interpretive challenges, difficult choices, and how researchers’ biases and goals shape findings. Concerned by the absence of communication with and to people with disabilities during the COVID-19 pandemic, we turned to the School of Public Health material archived at the University of Toronto to uncover preserved stories of previous public health crises. Using our research questions about communications as a case study, we document how researchers navigate archival absences, make decisions about what counts as evidence, and construct interpretations from incomplete records. We describe three critical methodological turns: re-orienting, re-encountering, and re-searching that emerged from confronting archives organized without disability as a category. Our methodology resists bounded chronology to trace new communicative connections across an array of material: letters, photographs, research grants, and public health lectures. We contrast the abundance of ableist internal departmental communication that is preserved in the archives with the illegible paucity of external communication to and about people with disabilities. We attend to our own embodied acts of communication: reading, photographing, pasting, labelling, and assembling seemingly tangential documents from across hundreds of boxes of archived material while simultaneously acknowledging the surveillance work in the library archive and our own preservation of some materials and procedures. Our findings about how disability is included as disease-like but excluded in many other ways in public health communication are inseparable from our methodological choices. What we found depended fundamentally on how we looked, what we decided counted as relevant, and how our commitments to disability-as-a-communicative subject shaped our seeing. This paper contributes both specific insights about disability communication and a methodological model for conducting reflexive archival research on marginalized communities.

## 1 Introduction

This article maps some of the methodological moves made as we sought representations of disability communication in the University of Toronto’s School of Public Health archive. By “disability communication,” we mean archived material that either communicates *about* disability issues or communicates *to* disabled people/people with disabilities. Finding a paucity of direct disability communication, this paper maps and interrogates some of the methodological contours involved as we pursued the meaning of disability and communication when it is not made directly present by the archived material and yet *must* be present given that the amelioration of disease and disability is foundational to Public Health. Our delineation of interpretive methods in archive work was guided by this question: “How might archival methodology investigate disability communication when direct and explicit communication with disabled people does not seem to be present?” For anyone concerned with the reach of public health, such a question is key.

Shifting from “archives-as-things” toward a sense of the “archives-asprocess” (Stoler, 2002) is how we supported our shift in analytical attention to what does not readily appear. We treat the archival *content* (what documents say and preserve) as part of what produces (and relies on) that which does not directly appear. Attending to what does not appear is further pursued by reliance on three key methodological moves which we map in this paper through a descriptive reflexive interrogation of our research activities: activities of re-orienting, re-encountering, and re-searching. That is, our interpretive disability studies approach made *re-orienting* to the apparent absence of disability a hidden presence and our starting point for *re-encountering* how public health, disability, and communication are given cultural form by *re-searching* other taxonomies of human difference and distinction. This framework also required us to be transparent about our own role in the ongoing process of archival meaning-making.

In keeping with our emancipatory research aim, we model a research practice that acknowledges rather than erases the subjective, embodied, political dimensions of knowledge production. This paper thus makes two inseparable contributions. Substantively, we explore historical patterns of communication with and about disabled people during health crises, revealing how disability has been rendered simultaneously hypervisible (as an assumed disease object) and invisible (as communication subject). Finally, we demonstrate how these findings emerged from specific researcher choices, theoretical commitments, and interpretive strategies. Every insight we present about disability communication reflects not only archival content but our decisions about how to read, categorize, and interpret that content.

### 1.1 Background

Our initial project had different beginnings. Concerned by the absence of communication with and to people with disabilities during the COVID-19 pandemic (Landes et al., 2021; Lunsky et al., 2022; Read et al., 2022), we turned to the past to explore that which preceded this time, to find once again disability “overlooked” (Ellis et al., 2024; Martin et al., 2022). The COVID-19 pandemic was certainly not the first health crisis that the world had experienced, nor was it the first time that disabled people were objectified as vulnerable but not necessarily oriented to as a communicative subject. Since our question centred on communication with disabled people or about disability and health issues, we next asked who was typically responsible for communicative acts during health crises. Again, the answer seemed self-evidently related to public health. The Institute of Medicine defines public health as “what we, as a society, do collectively to assure the conditions in which people can be healthy”.^1^ Clearly, public health education and agencies were part of the communication landscape of epidemics, health, and (we assumed) disability.

To explore this question, we turned to archived materials from 1918 to 1970 related to the Dalla Lana School of Public Health at the University of Toronto to uncover preserved stories of past public health crises regarding disability matters. We chose this time frame because 1918 marks the beginning of a deadly flu pandemic, and 1970 marks a time when the internet had not yet transformed mass communication. Further, we confined our research to one university unit (Public Health) so that we could focus more on how disability appears, without the need to conduct comparative work across units and disciplines, universities, provinces, or countries. Put differently, with both a restricted time frame and restricted location, we aimed to focus on the “obvious” need to communicate about disability by an organization that we assumed would be involved in doing so, or at least teaching people to do so, thus our turn to public health at the University of Toronto.

Our initial research project^2^ was based on an immersion at the Public Health archives at the University of Toronto, where we searched for direct communication either about disability or to disabled people. Theoretically, our work was oriented by a disability studies approach that is informed by interpretive sociology and the humanities. Our search began with the assumption that, irrespective of biomedicalised definitions of disability, disability exceeds being merely an individual vulnerability with associated risks – disability is part of human subjectivity and is tied up with the promise, possibility and limits of our interrelatedness with and through cultural communicative expectations. Our proposed archival methodology was ecological rather than historiographic, drawing from multiple sources from this one unit and focusing on forms of relationality while not focusing on the historical movements of a general public health crisis response (Scott, 2014; Stinchcombe, 1965, pp. 169–180). We reasoned that this ecological focus would allow us to pursue how conceptions of health, communication, and the public, including disabled people, were being co-constituted via the environment and logics of public health engagement as manifest at a major Canadian university.

### 1.2 Initial methodological beginnings

We, Aparna and Tanya, PhD student and supervisor, began work in August 2023, having received research funding from the newly established (2022) Institute for Pandemics at the University of Toronto (renamed on November 4, 2025, as the Institute of Health Emergencies and Pandemics – IHEP; and with conceptual support from Dan Goodley’s, Disability Matters, Wellcome grant). We proposed to the Institute that our research would explore the variegated constellations of meaning that crystallize around the words “disability,” “communication,” and “health.” We also proposed to identify what was missing from the archive, allowing us to demonstrate how communication and conceptions of disability-as-diversity matter or do not matter to public health education and its initiatives. Such an approach, we believed, would uncover more nuanced articulations of disability buried within the archive. Our initial goal was emancipatory – how to better conceive of disability issues and the communication of health matters with disabled people to enhance the possibility of life when faced with the next health crisis. Exploring and uncovering this sense of disability communication during pandemics could demonstrate the essential need for diverse forms of communication, thereby influencing the development of disability communication protocols not only for future pandemics but also for other health crises.

At the archives, we chose records related to university governance of public health/hygiene, as well as to faculty members, their research, and a variety of university educational programming decisions and documents. We sought out any way that “disability” crystallized, that is, made an appearance in the archived material. This material ranged from exam papers, meeting minutes, annual reports, and lecture notes to research grants and travel journals – the many minutiae of the school’s past ranging from 1918 to 1970. A time frame that began with a well-known pandemic event and ended before a variety of electronic communication transformations took place in public life. We aimed to engage with the archives to map the ways in which disability had crystallized, that is, been made present in the material. Absence, as we will now show, was the clearest depiction of disability that we received.

### 1.3 Taking a (methodological) turn

The archives yielded little evidence of the presence, much less involvement of disabled people in previous health crises. For example, serums for polio were developed for an imaginary referred to as the “ordinary Canadian child” (Hastings C.J., 1918 cited in Bator & Rhodes, 1990, p.2) while in a letter written by Dr. Charles J. Hastings, Toronto’s Medical Officer of Health from 1910 to 1929, public health communication was addressed to other imaginaries such as the “man on the street” or the “housewife in the humblest home” (Hastings C.J., 1918 cited in Bator & Rhodes, 1990, p.2). We found ourselves in an archival space where public health archives seemed to contain aspects of the material histories of science, medicine, health, and histories of national and international funding, but few signs of disability. These histories were interwoven with stories of capitalism and colonialism and entwined with the voices of leaders in health and governance. Where, in all this plenitude, were we to find disability communication?

Till today, disability remains a repressed yet omnipresent feature of the “race, gender, class” trifecta but is still often left out of various conceptions of equity, diversity and inclusion initiatives. Even though disability, in the form of embodied differences, is likely to be the most common feature in any large arbitrary grouping of people, disability has still not come to manifest as a common way to start to imagine the “public”. However, this absence of disability representation in the archived material did lead us to a significant dilemma.

How could we learn lessons from past pandemics to improve future disability communication when there was little to no archival evidence of such communication in the first place? Moreover, even when disability did appear directly, it was not through any kind of mutual subjective recognition caught in the weave of communicative expectations (for better or worse). Instead, and more troublingly, it was depicted objectively - as a problem in need of elimination and/or amelioration.

At this point, we reminded ourselves of Alia Al-Saji’s suggestion that any fractured past of colonial history asks for a “critical hesitation” (Al-Saji, 2018, p. 346). Critical hesitation involves deliberately slowing down to attend to the interpretive act itself. We were looking for any appearance of disability (certainly an interpretive act), thus we needed to question our initial readings, and acknowledge what we were looking for. If we were not to find “disability” directly addressed as a communicative subject, what else might evidence support for the presence of disability? Rather than treating the ambiguity of appearance as a problem to be defined prior to approaching an archive, we treated ambiguous expression as valuable information about the nature of archival knowledge itself. And thus, we hesitated. We paused in order to consider not only that the archive served to split us off from what we were eager to find, namely disability communications, but that this absence itself represented further fractures that might be grappled with through a critical hesitation.

Facing the fracture between what we were seeking and what we were finding required us not to rush to bridge it with a justification or an explanation, but instead to open some “breathing room” amid these different sets of interests. This hesitation returned us to the sense that it was not surprising that disability communication was missing, and that the missing might be visible only to those positioned within a disability studies approach. We hesitated here, allowing ourselves and our work to expand our thinking from the what of the search to the how of the search. How would we re-search the archives? *Re-searching* the archives needed us to first *re*-*orient* to disability studies - not just as a static theoretical positioning standing behind our work - but as a living, breathing way of immersing ourselves. Here, we drew upon Minich (2016) and Schalk (2017), who call upon the need to understand critical disability studies as a *methodology*. In other words, occupying and writing from a position where disability studies functions not just as a theoretical framework or area of scholarly conversation, but as a guide for conducting research, Schalk writes that by “Following Minich, we can understand critical disability studies as a method, an approach, a theoretical framework, and perspective—not (exclusively) a study of disabled people. One can study disabled people and not be doing critical disability studies, and one can be doing critical disability studies and not be directly studying disabled people” (2017, p. 2). Putting these insights into conversation, we experienced a *re-orientation*. It was not a direct address to disabled people that would remain our primary concern; it was that *disability issues and disabled people “had” to be there all along*, and it was crucial to uncover how (Manning, pp. 52-53 as cited in Titchkosky, Healey, & Michalko, 2019, p. 135).

This led us to consider how our work could centre solidarity with disabled communities and accountability to them. In other words, we began to frame disability communication in the archives as a methodology not merely meant to pursue the absences of disability communication but to *address* the significance of exclusions and interpretations of communications that ignored or, at best, only sought to prevent disability. Instead of a continuous and often vain search for disability communication, we now entered and absorbed the failure of the records to enact attention to disability in public health.

Embracing the discomfort of archives as indexes of absences and omissions, we asked different questions: what was conserved? What was destroyed in order to conserve? By attending to this fugitive underbelly, our methodology now illuminated the dualistic paradox of conservation and destruction upon which archival stories rely. We found Judith Butler’s reflection on recognition helpful. Butler suggests that recognition

“is at once the norm toward which we invariably strive … and the ideal form that communication takes when it becomes a transformative process. Recognition is, however, also the name given to the process that constantly risks destruction and which, I would submit, could not be recognition without a defining or constitutive risk of destruction” (Butler, 2007, p. 133).

Again and again, we came across archived stories where disability was recognized as something to be prevented and destroyed through its paradoxical conservation as a preventable disease or threat. Disability was thus rendered as the unwanted outcome of ill health, but not as a subject with whom ways should be found to communicate about matters of health, life, and death. Disability was nowhere to be found as a “public” in need of attention. Just as we moved from searching to *researching*, we now found that attending to these stories required us again to *re-orient* methodologically.

## 2 The first turn: Re-orienting to disability communication by attending to destruction, conservation and the other

### 2.1 Destruction

We found that disability, manifested in the archives as an abstract concept by taking shape as an uneasy sibling of disease. Throughout our chosen research time frame (1918 – 1970), disability was conflated as disease, made into an inseparable pair; for instance, funding was made available for “the prevention of disease or disability” (University of Toronto Archives (hereafter UTA) B77-0047/002). The appearance of disability, especially when placed in the family nexus of disease, was paradoxically marked by the simultaneous interest, even plans and programs, for it to disappear. Take the example of this National Health Research Grant application form of 1966 that anticipates the dissolution of disability even before people with their own relations to disability have fully arrived.

**Fig. 1:**
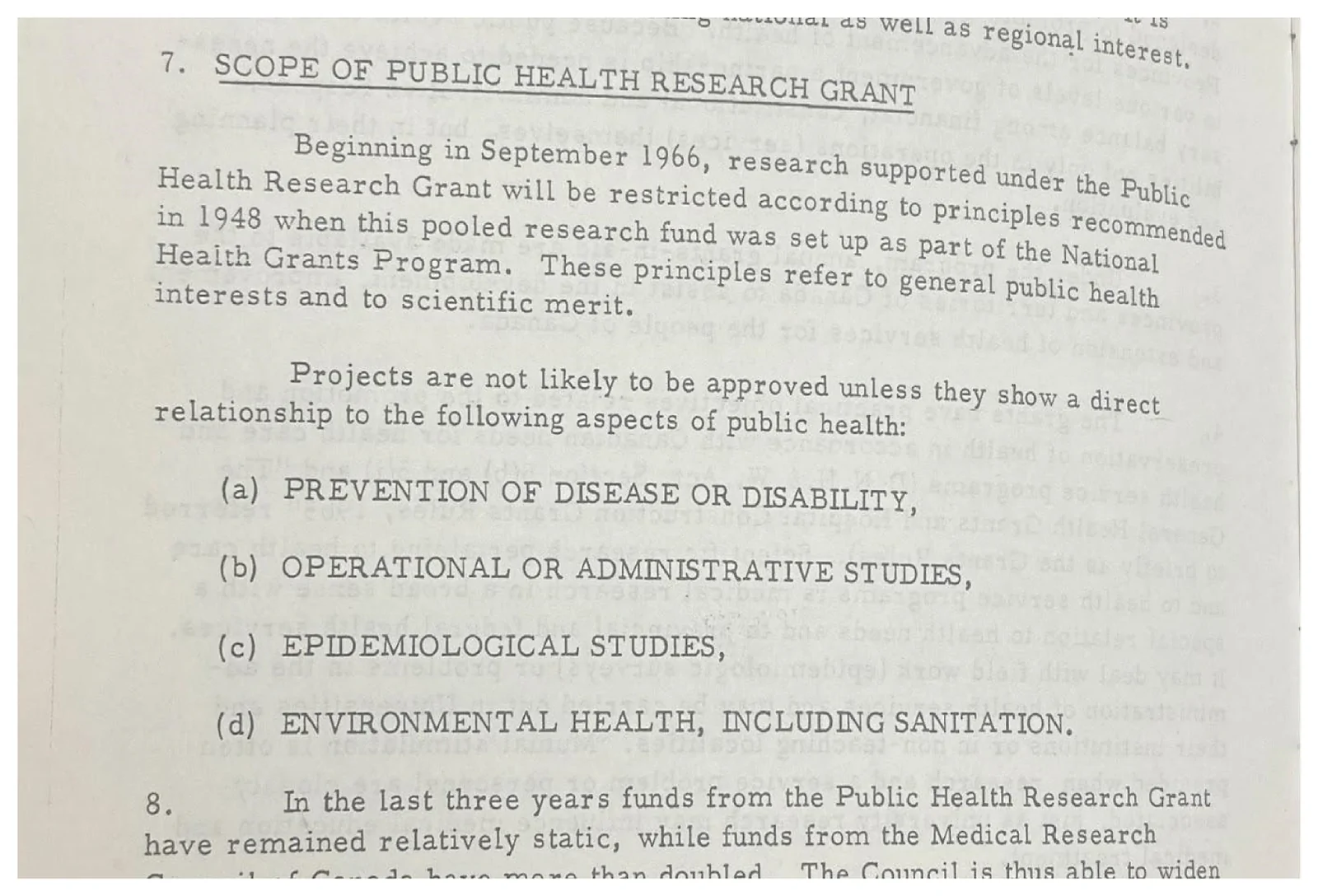
National health research grant application Form, 1966 (UTA. B77-0047/002).

Those who apply for research money from the state, announces this application form, are “not likely to be approved unless they show a “direct relationship” to the “prevention of disease or disability” (Fig. 1; UTA. B77-0047/002). While it was difficult to find any direct address of disabled faculty, staff, students or “the public” in the public health archived material, the need for research to “directly” prevent disease or disability is made explicit. The lack of representation of disabled and disabled people in this public health archive, along with the scantness of external communication to and about people with disabilities, was not necessarily surprising. What was surprising was the unending omnipresent indirect sense of disability as only an unwanted *consequence* of ill health.

### 2.2 Conservation

Even when written about and present, disability was often enacted as a sort of marginalia, given minimal space, addressed as an unwanted entity (defined via its negation), within the copious yet measured and orderly affect of official curricula or grant application instructions. Pausing to attend not just to content or the absence of content, but also to form and to textual rules of placement and reference allowed us to attend to what kind of disabilities were imagined, feared and considered out of place in the system of public health. Consider, for example, these accounts of research that the 1966 Public Health grant “encourages:”

**Fig. 2:**
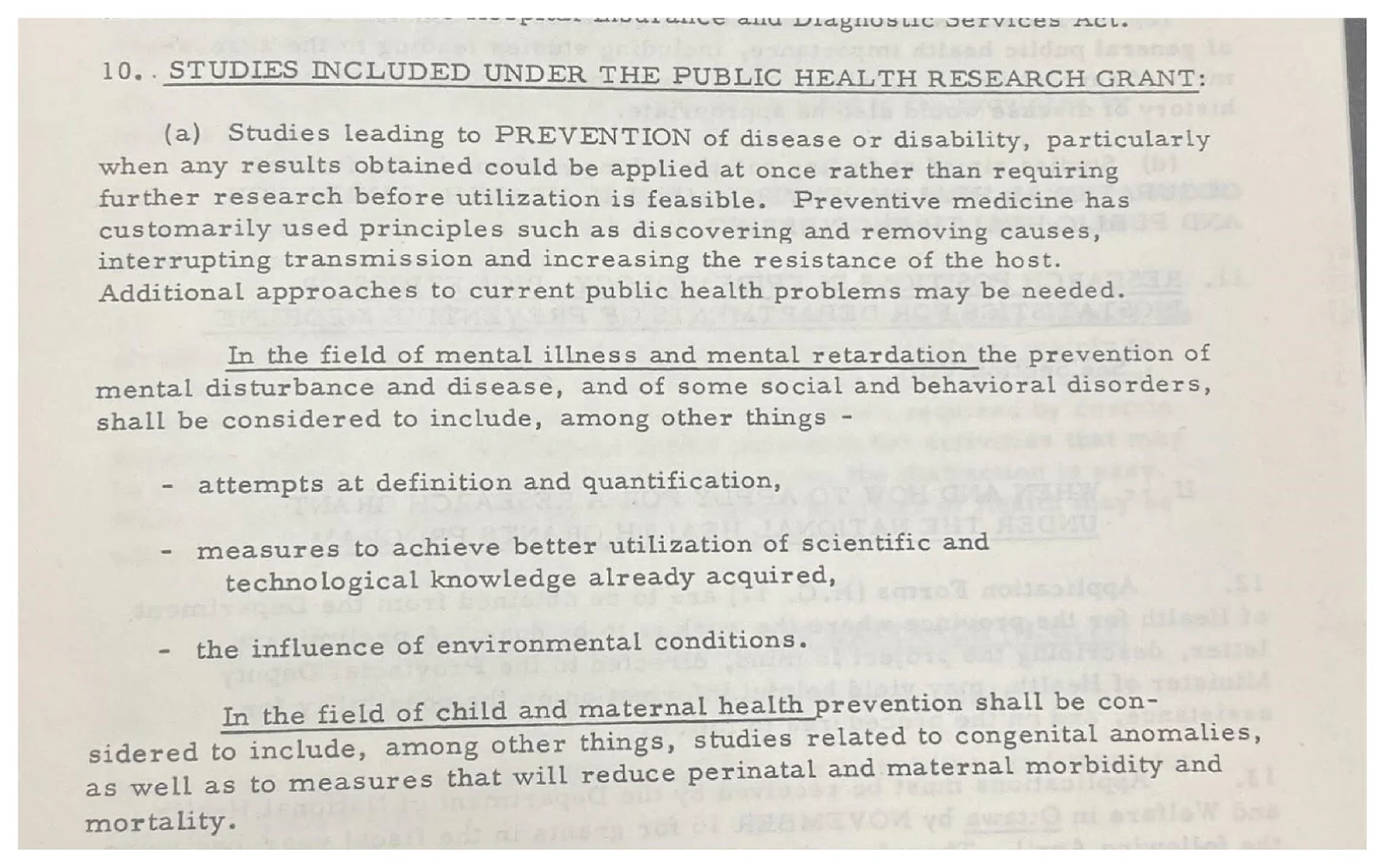
1966 public health grant UTA. B77-0047/002/ETC.

“Studies leading to PREVENTION [*in capital letters]* of disease or disability […]” including the “prevention of mental disturbance and disease, and of some social and behavioural disorders” will be funded, announces this grant rubric. The rubric goes on to say that funding will be contingent upon the said funded research’s “attempts” to “define” and “quantify” “mental illness” and “mental retardation” (Fig. 2; UTA. B77-0047/002), even though disability is already defined within the Grant announcement as disease-like and in need of elimination, amelioration, or control. Nestled in a section on child and maternal health, we found, amidst attention to maternal death and morbidity, a quiet demand for research that seeks the prevention of “congenital anomalies” (Fig. 2; UTA. B77-0047/002). While the concept of disability is rarely, if ever, discussed directly, its prevention, that is, a future absence of disability, is discussed and detailed as a phenomenon so objectified that it can be found, defined, and its quantity measured.

### 2.3 The creation of the Other

In his well-known writings on the archive, Derrida posits the idea that archives materialize at the breakdown of memory (Derrida, 1996). The impulse to conserve archived material is founded upon the desire to forget. That is, remembering requires attending to something by not attending to something else. The production of varied communicative acts in the public health archive did not emerge in isolation. Still, it did enact the desire to forget disability as being anything other than disease-like (which makes forgetting disability as life-like necessary). The failures of the archives in recovering and remembering disability as life and thus a communicative subject nonetheless support the relentless valorization of the medical achievements of public health programs. When we read the material through a disability studies framework, it became evident that this valorization could only be fulfilled by denying the disabled “public” any kind of complex representation.

**Fig. 3:**
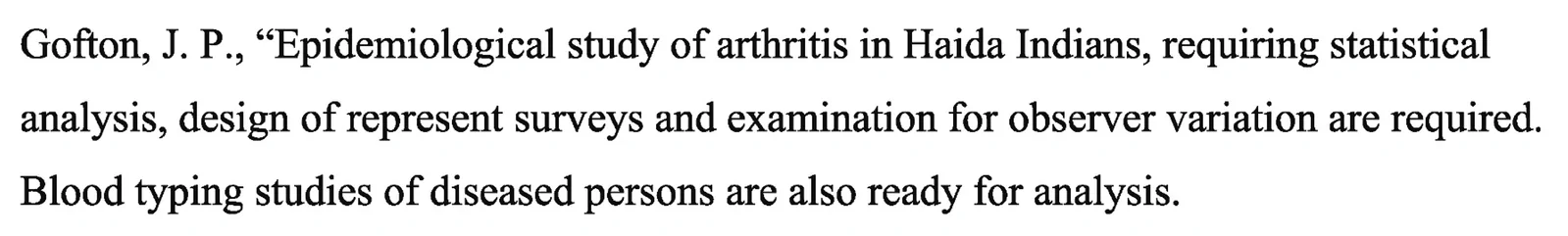
Haida project (UTA b77-0047/002).

Consider the following. In 1966, the same year as the research grants above, the school hired a Miss Brenda Jane Morrison as a biometrician with the aim of addressing the “increasing complexity” of human biological and environmental forces and the “planned evolution” of preventative medicine. The advancement of medicine is valorized but what of the public, the people subject to these advancements? The university needed a mathematician to create mathematical models of disease distribution and epidemiological analysis of “population groups” of various on-going research projects. One such project, depicted below, refers to the taking of blood samples from Haida Indians to measure levels of pollution (Fig. 3; UTA B77-0047/002).

**Fig. 4:**
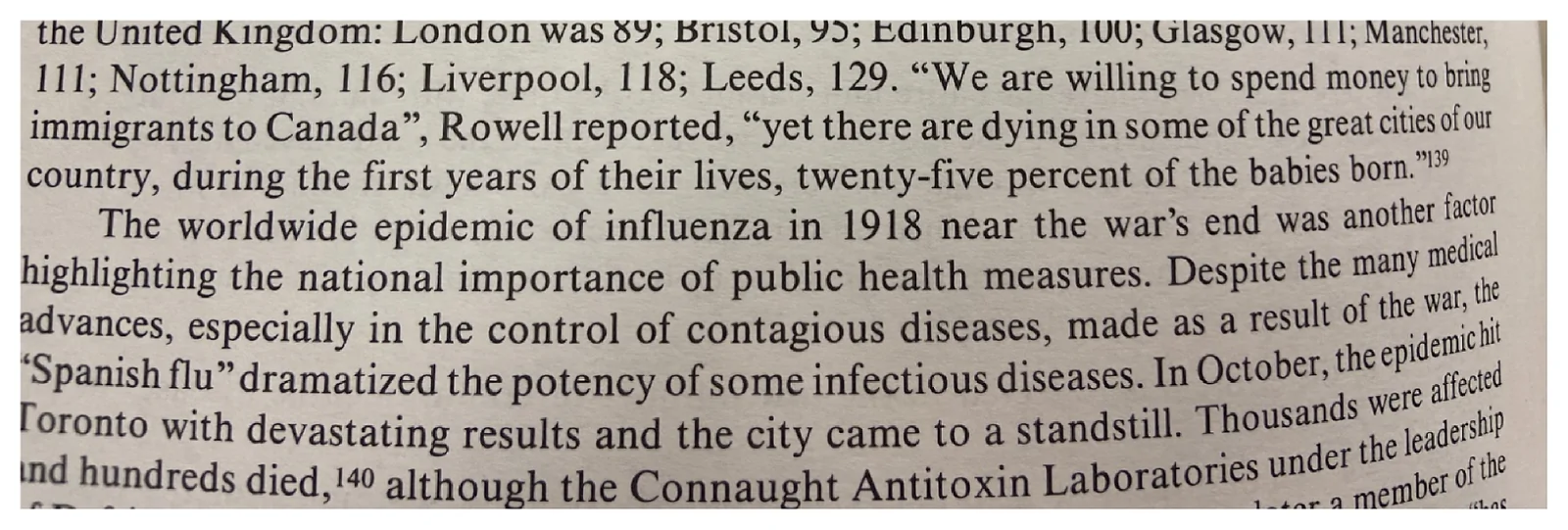
Description of 1918 Public Health Crisis Bator & Rhodes, 1990. *Within reach of everyone: a history of the University of Toronto School of Hygiene and the Connaught Laboratories*. Vol. II. The Canadian Public Health Association, Ottawa, Ontario.

There are a wide array of descriptors present in a relatively short text that was used by the school to justify the need to hire a mathematician/ biometrician and that would serve as a material description of Miss Brenda Jane Morrison’s future work. The descriptive signifiers, “Haida Indians,” “population groups,” “blood types,” and “diseased persons” rely on the self-evident formation of the “public” in public health. How? By forgetting the always already presence of disability, reconstituting disability only as a potentiality - as the background threat of arthritis or illness and disease consequences, potentially present but hopefully absent. Similar communications, such as the one below, emphasize the “national importance of public health measures”. Such stridency needs a foil and is thus provided one via the silence of the homogenized “thousands” who were “affected” and the “hundreds [who] died” within various health crises, such as the “worldwide epidemic of influenza” that struck the city of Toronto in October 1918 (Bator & Rhodes, 1990 p.2; Fig. 4).

Recognizing public health education narratives could not be accomplished without a corresponding conservation of disability as a disease or an unfractionated threat to be destroyed.

The simultaneous destruction and conservation of disability as disease allowed the construction of varied and plentiful descriptions of how public health could be endorsed and made relevant.

Reading the presence of communication through its absence was thus an important methodological *re-orientation* through which we attempted to reveal some of the communicative interests of the archive. The attention we paid to destruction, conservation and the creation of an Other as part of our methodological re-orientation described above thus led us to read for the lavishness and detail of such communication *through* the often-illegible paucity of external communication to and about people with disabilities. It is in this sense that the archive communicates that disability is a threat animating public health education endeavours - even though nowhere are disabled people directly addressed.

We began to think about who was addressed by the archived material and who was not; of those addressed, did they form a “public” or some other imagined group of public health interest and activities? We also began to think about *how* we knew. So, we returned to the University of Toronto public health archives with our guiding question somewhat redesigned: What did it mean to be present but also to be silenced in the archives? And *how* was this communication enacted? How did both this presence and absence take place simultaneously?

We realized that the interpretive work we wanted to pursue – understanding how disability communication can be an important tool for historically contextualizing the work that public health projects and education have carried out – could not exclusively depend on objectified, defined content. Instead, our methodology needed to expand to reconceive the idea of an archive search as an *encounter* that would place the act of collecting and the collectors centre-stage rather than the contents of the collection. This methodological expansion allowed us to *re-encounter* and engage with groupings and documentation that were forceful yet unsure, authoritative yet hesitant, and characterized by both polyphony and silences. We needed to think about the affective response that the documents elicited in us as researchers while reading them. In this, we came to view our next methodological step of re-encountering as an interaction between *access* and *affect*.

## 3 The second turn: Re-encountering and the act of unboxing: putting access and affect in conversation

### 3.1 The question of access

Some work in archival studies focuses on how archivists are active participants in what is available for excavation (Koh, 2014). Re-encountering the archives meant, for us, putting the question of access and affect in conversation to document our time at the archives. “Access,” writes Titchkosky, is a “way of bringing life to consciousness, a form of oriented social action, and a way of relating to people and places” (2011, p. 3). There are a variety of ways that access to the archive raises issues of consciousness, social action, and relationality. For an encounter with people and places of disability communication, our meetings in the archive were often conflictual in different ways, unevenly accessible, and subject to surveillance. We were able to financially engage in this research project because of a grant from the newly formed Institute for Pandemics (now IHEP), which funded our archival project on disability and pandemics – a privilege not available to many disabled people without academic affiliations.

Next was the question of physical access. We would climb up the steep steps of the Thomas Fisher Library (the more accessible entrance was tucked away at the back via a long ramp), jostle for space in the tiny, cramped cloakroom of the building and wrestle (usually unsuccessfully) with recalcitrant locker keys. The staff at the turnstile inspected our laptops, making sure everything was in “clear plastic bags”. Extras, such as pens, hot drinks, or food were prohibited. Once in, we signed multiple research agreements promising to protect the confidentiality of the owners of these ephemera. Finally, we find ourselves in the reading room, a windowless room that provided tables, chairs, a giant scanner, and fluorescent lighting, a room that was practically impossible for a wheelchair user to enter. We sat there carefully handling grayed and yellowed paper frayed around the edges, little of which was digitized, available online, or accessible to a researcher with visual impairments, conscious of the many disclosure agreements we had signed and of our requisition forms filled in by hand in duplicate. The absence of disability was made manifest through these various procedures of archival access.

### 3.2 The question of affect

We came to our encounters with the archives, bringing our different nationalities, cultures, races and career stages with us. A tenured professor and her PhD student. A disabled human and a disability caregiver. White and brown. All these apparent differences and, yet, we soon decided that the large table in the centre of the room was “ours”. As the brown boxes we had requisitioned were wheeled out on a dolly, we would silently turn to each other, our bodies and minds palpably registering simultaneous excitement along with a familiar anticipatory feeling of omission. What would we not-see today?

Methodologically, it was not enough to view the archives as complicit in creating stories of the exclusion of disability communication – our own agency that lay in our relationalities to each other and in our own intersecting and opposing identities also impacted the reception and perception of our sometimes conflictual, sometimes emotional, and sometimes amused unboxing of disability and public health. We spent months scanning through pages and pages of books, letters, manuscripts, exam questions, and lecture notes. We often spoke of the scarcities and silences of disability communication, but in this act of re-encountering, we realized that these scarcities and silences around disability communication were, after all, contingent. They took shape only through our agential act of looking and reading, coming into existence when *we* looked for them.

### 3.3 Access & affect

When we put the question of access in conversation with the question of affect, we realized that our encounters were both contingent and anticipatory. Our project had commenced with this thought: the promise of a future pandemic was the reason we drew on the past. Alison Kafer writes of how disabled people “live in a kind of anticipatory time, scanning their days for events or exposures that might trigger a response” (2013, p. 31). We, too, approached each brown box with a sense of affective anxiety tied to this anticipatory sense of time, a distressingly easy familiarity with partial, problematic, harmful, inaccurate, and/or absent representations of disabled people in the world, the archives and potentially our disabled futures as well. What would we find today? One of us is dyslexic, and the other is a mother to a young child with multiple disabilities, including Down syndrome and autism. The plentiful references to “mental health,” “mental retardation,” and “the problems of handicapped children” did not simply belong to examiners and public health officials of the past but touched us as researchers, in the here and now.

Re-encountering the archives allowed us to become aware of the inherent ableist and normative ways in which one is expected to behave within archival spaces. The archival room was marked by the quiet turning over of pages. Talking was not forbidden, but an unwritten rule seemed to ask us to talk in little above a whisper. Re-encountering our laughter at a photograph or how we reacted to it made us realize we performed acts of interpretation and made meaning of them through the constraints and possibilities provided by our lived contexts of the archives’ organization of space and time. Both access and affect were our guides here. We found that a disability-studies-informed methodology that desired to center the complexity of disability communication also needed to account for the complexity and unsettling of our own responses to the material. Recalling the writings of Minich and Schalk, who helped provoke our re-orientation, our affective responses needed to form part of our methodology because, whether anticipatory or resultant, the affects that were evoked by the material we had access to *made* records significant inviting further attention. Our own lived experiences of disability, access, aspirations, fears and distrust became the forces that, at least partially, shaped the archives and what they offered.

But *what* material would we attend to? Following a critical disability studies methodology, how could we continue our search for the place and meaning of disability in the public health archive? The question that arose now was how to return and methodically engage with both what we were and were not finding — what we refer to as *re-searching* the archives.

## 4 The Third turn: Re-searching disability communication via the fonds and other taxonomies

### 4.1 Re-searching the fonds

Unlike libraries that are catalogued via subject, the archives were categorized via *fonds* - in effect, groupings of documents collected by a single person, program, department, or institution. The concept of fonds originated in France in 1841, when the role of an archivist was defined as bringing together “all the deeds [which come] from a body, an establishment, a family, or an individual” (Thibodeau, 1993, pp. 256–258). Canadian archivist Terry Cook defines fonds as “an intellectual construct.” “The fonds,” he writes, “is thus the conceptual ‘whole’ that reflects an organic process in which a records creator produces or accumulates a series of records which themselves exhibit a natural unity based on shared function, activity, form or use” (1993, p. 33). Central to the idea of fonds is a belief in the sanctity of the origin of collections or their provenance to represent the body from which they arise. This, in turn, is based on the belief that maintaining a collection’s origin also, at least partially, preserves its context (Pearce-Moses, 2005). The concept of the fonds has thus widely influenced the idea of creating collections of documents that are “naturally” and/or “originally” accumulated around a theme, an organization or a person, even a country (Thibodeau, 1993, pp. 256–258).

This belief in the part being a sufficient representation of the whole, as if that part arises organically or naturally from the (whole) body in question, is a common epistemological naturalization within the contemporary Western milieu of knowledge production (Hantel, 2018; McKittrick, 2015). This way of forming knowledge or an adequate representation is part of the contested terrain within disability studies and other forms of critical scholarship that question the social organization of naturalized versions of knowledge (Canguilhem, 2022; Titchkosky, 2003; Titchkosky & Michalko, 2009).

**Fig. 5:**
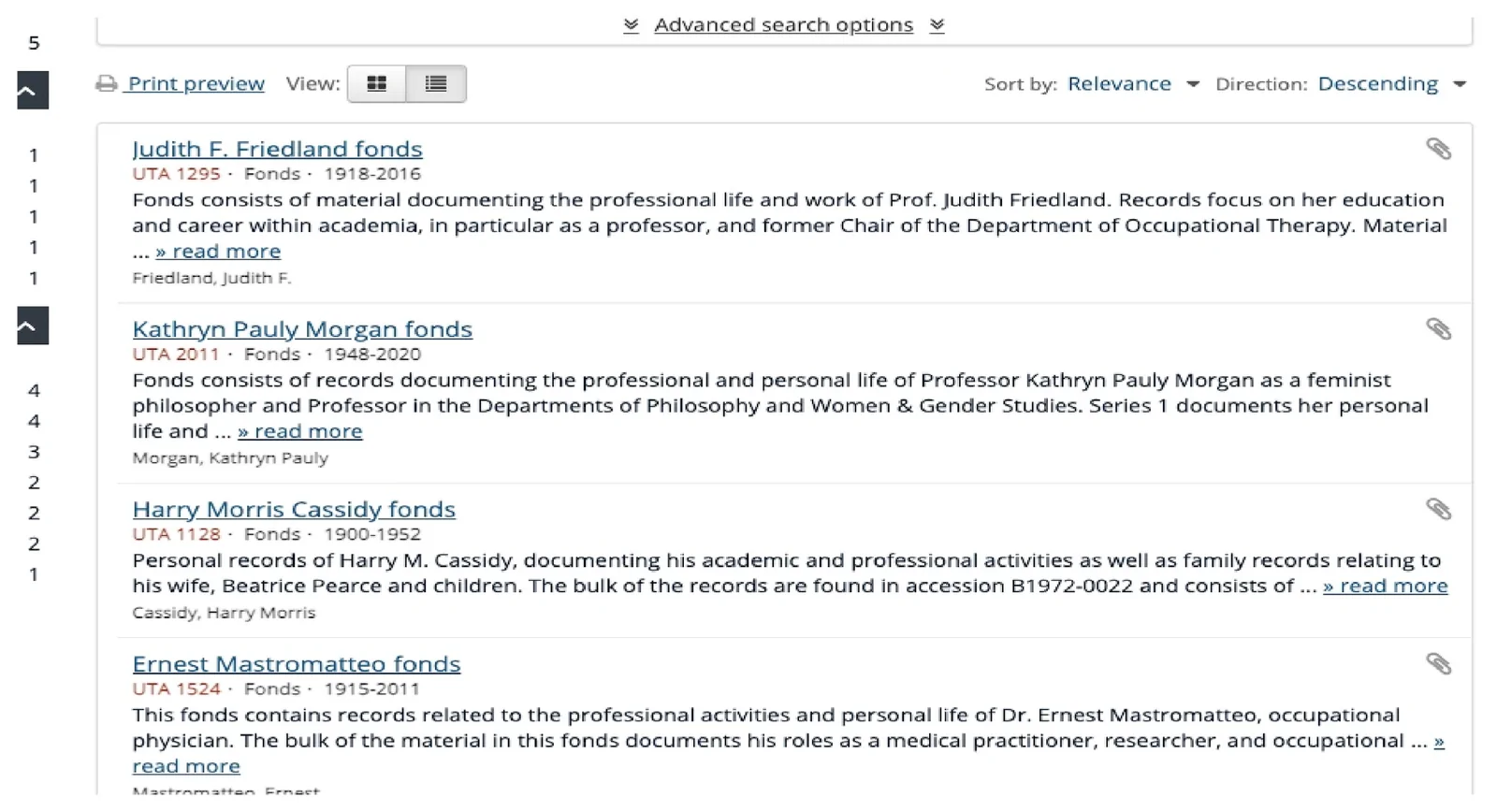
Screenshot of fonds discover archives. Accessed April 10, 2025. Url: https://discoverarchives.library.utoronto.ca/index.php/informationobject/browse?repos=391&query=public+health+AND+disability.

In our various searches, for instance, we encountered fonds sorted by an index of academicians. For example, an electronic search of online fonds with a key entitled “PUBLIC HEALTH AND DISABILITY” returned this (Fig. 5). The names of directors, deans and others were listed to label and sort the fonds that collected the ephemera of a career in academia that had some relation to public health. What that relation was required further engagement with the fonds. When we read *with* the fond, we would encounter documents that referred to “communication,” others to “education,” and yet others to the “practice” of public health (UTA A75-0029/003). Despite prevailing ideas of fonds as a “conceptual whole”, the rules of classification that governed the creation of fonds seemed to be a fragmented and unruly affair, cropping up and disappearing seemingly at random. In other words, how the part is made to represent the whole or comes to represent the whole is far more arbitrary than it may first appear. Once we moved beyond personal and professional fonds of individuals who had some relation to public health and into those who helped establish, govern, or play a decisive role in the unit’s development, there were solidified or institutional terms such as “education” and “community” listed as entries, but conceptual items such as “disability” and “communication” were largely missing from the index. Therefore, the conceptual construct of the fonds imposed a discursive limit by providing no easy way to reveal the interconnectedness of our interests.

Drawing on the work of historians working to uncover the communicative acts of marginalized pasts, we realized that we too needed to make cuts or read *against* the fonds (Benjamin, 1999; Cook, 1993; Hartman, 2008; Stoler, 2002; Trouillot, 1995).

**Fig. 6:**
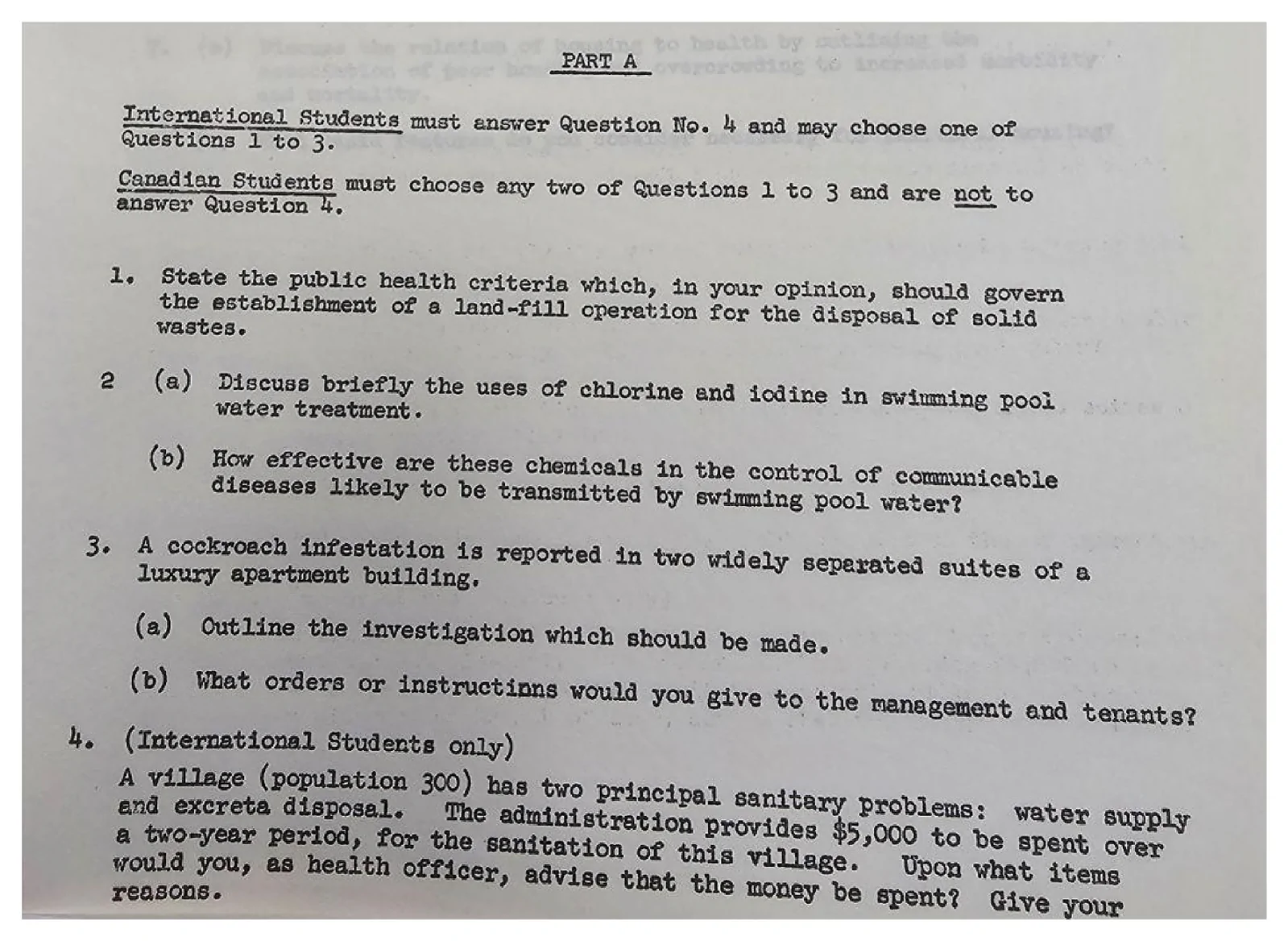
1962 sanitation exam UTA. Papers of milton Brown. B1977-047/005/ETC.

For instance, in the Sanitation Exam (Part 2) held in 1962, students were asked about the “use of chlorine and iodine in swimming pool water treatment” (Fig. 6; UTA. B1977-047/005/ETC). When we read this against the examination paper from the same year, which included additional questions for international students, the question posed to international students asked for a description of “water supply and excreta disposal” in “a village” (UTA. B1977-047/005/ETC). As we read this, we wondered aloud, “What would Foucault make of this?” According to Foucault, examinations represent a “normalizing gaze, a surveillance that makes it possible to qualify, to classify.” Examinations establish “over individuals a visibility through which one differentiates and judges them” (Foucault, 2020, p. 174). This exam, like many others, was found in the professional and personal fonds of director Milton Brown, collected into a seemingly natural, organic whole and listed under the name of a previous academic leader at the school. We needed to *re-search* to uncover how archived material and subject were inextricably linked through the classification and objectification of what constituted a domestic problem and what constituted an international one. This process of objectification was carried out via the routinized discourse of examination, through which living human students were transformed into “Canadian” or “International” cases with different problem areas to be tested. While this examination does not explicitly mention disability, it reveals how public health training communicated racialized and ableist norms about bodies and environments. Canadian students, for example, are examined on external disease threats (cockroach infestation) in an exclusive context (luxury apartment building), whereas international students are asked to deliberate on human excreta, a problem in, of, and for all humans but affecting a small village with limited resources. These assumptions shaped how future public health officials would understand which populations required which kinds of interventions and at what cost.

Re-searching the fonds using critical disability studies as a methodology prompted us to hold categories of concern, such as racism, disease, illness, and disability, together within “embodiment” at the forefront (instead of as a taken-for-granted background) in order to decenter unexamined conceptions of whiteness and able-bodiedness that inform the education of future public health practitioners. The debility embedded in conceptions of what might threaten health comes to the fore when re-searching to determine where disability must be. That is, the disability studies framework for re-searching the fonds allows for a reading that straddles and maps the multiple imaginaries that connect swimming pools and sewage systems, making the question politically and racially charged. Re-searching the fonds was to acknowledge that these “naturalized” collections listed under a single academician constituted multiple and varied forms of subjectivity that divided and differentiated domestic and Canadian future public health officials, and how they would be examined.

### 4.2 Re-searching other taxonomies

While it was tempting to dismiss the idea of fonds as an artificial archival classificatory system that needed to be read against, the situation was more complex. It was not as though no other unpublished taxonomic system of classification existed *within* the fonds. On the contrary, public health documents, we realized, were themselves prolific and enthusiastic producers of taxonomies, that is, social categories – the “chronically ill”, “the aged”, those with “mental defects”, those with “cerebral palsy”, “mental hygiene” or the lack of it (UTA. A75-0029/ 003).

**Fig. 7:**
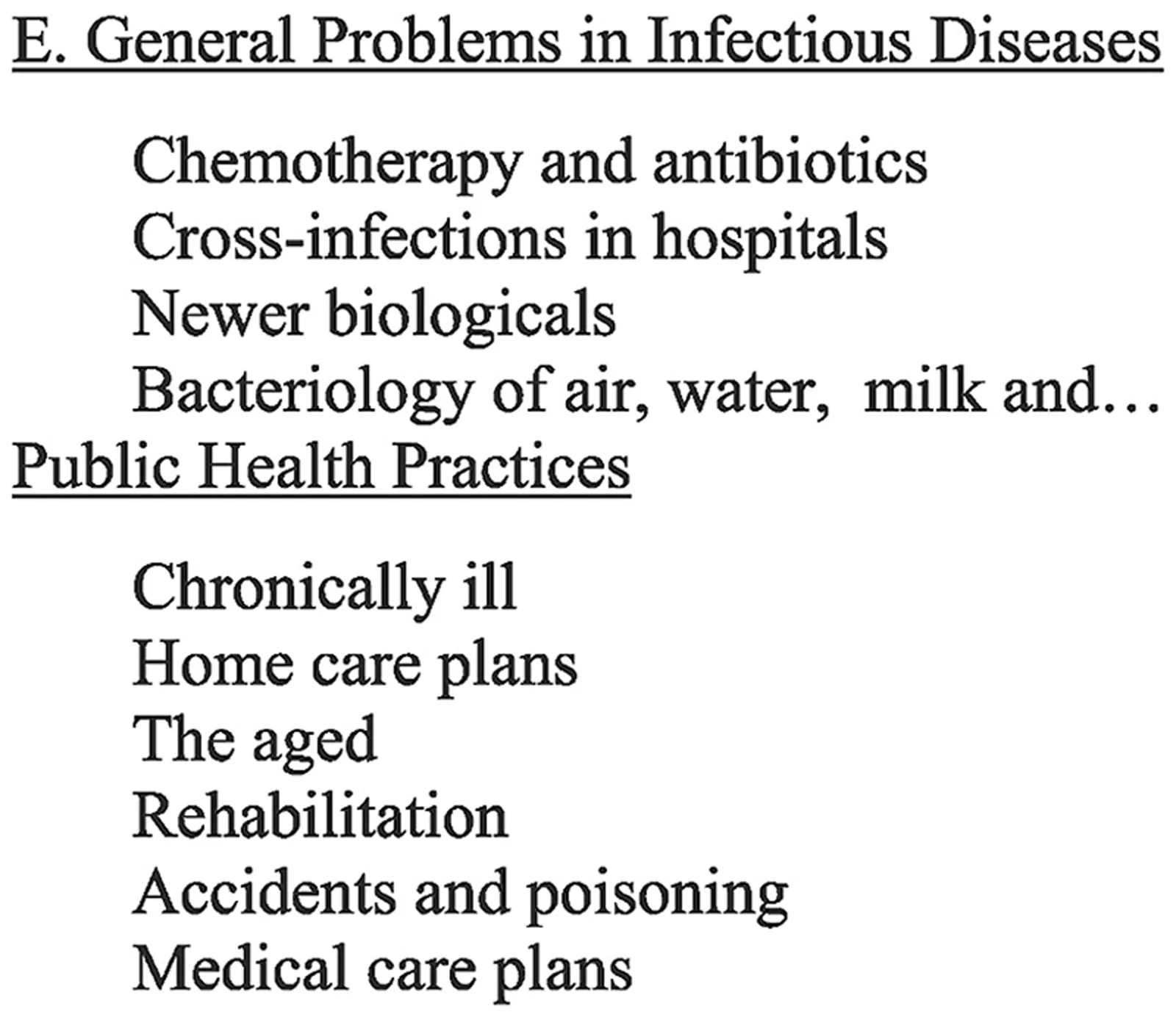
Public Health Syllabus 1952 (UTA. A75-0029/003).

In the below course curriculum document outlining the details of a public health syllabus in 1952, the “chronically ill,” “the aged,” and those in need of “rehabilitation” are listed under a course category entitled “Public Health Practice” (Fig. 7; UTA. A75-0029/003). *Researching* within the fonds, we realized, necessitated more than ever the need for a critical application of the political aspects of language. We paid attention - not just to content but to form. What was the genre, or which kind of documentation did we find these references in? What was the register or tone? What point was repeated, and what was said in asides or throwaway sentences? And how was this relevant methodologically to disability communication? As part of our methodological reorientation, we decided to study the making of the phenomena. Accordingly, the complicity of the archives in how it selected practices and simultaneously excluded the humans that make up these social categories impacted the structure of what was left behind in the shadows to be worked on.

Disability is, of course, the shadow figure throughout all of “Public Health Practice”, whether in illness, aging, or rehabilitation. Disability’s absence from direct address still helps to produce disability as the threat it is assumed all along to be, animating public health education. Such possibilities of communication existed, strewn across the pages of the archives, suggesting to all involved that the avoidance of disability is key to public health practice. *Re-searching* the communicative productivity of language to fashion the various interpretive possibilities of social categories revealed lacunae but also produced surfeits that exceeded the demands of disability communication proof.

## 5 Lessons learned or the risk that must be taken

We outlined above how an initial preoccupation with direct, explicit records that presented evidence was replaced by a sense that disability must be present, and thus a need to turn toward that assumed reality. We argue that our three turns of re-orienting, re-encountering, and researching drew our attention to and created space to think through the absences and presences of disability in public health. What, for example, happened to disabled people during this salmonella outbreak or a typhoid epidemic? The archives would have us believe nothing happened, since outbreaks and epidemics are mentioned, but the people most affected by them were not. Our role as disability scholars working with archived material was to document the lack of evidence, presenting it as evidence of a meaning made of disability, such that it would appear as a kind of disappearance. Our representations of these apparent absences could become evidence of another sort: evidence of the disability representation that did not make it directly into the archive. What concept of disability remains illegible, or even ineffable, resisting explanation or description?

Disability was everywhere, potentially present, even likely present, yet not figured as a character with which direct communication would be had. This sense of both in and out, in as an object of disease but out as a full subject worthy of interaction, reflects disability as a partial participant encased in liminal positionality. The life of disability is given shape by the public health archive as the social symbolic for “all that needs to be avoided” and “not life.” It is this conceptualization of disability, at least partially made for and by the public health archives, that health communication practices and protocols still need to learn to acknowledge and address today. Our methodological turns allowed us to refocus our gaze on what is missing, to consider the systems of power and devaluation that produced certain types of records, and to examine how those values became embedded in archives, further demonstrating the complexities of absences (and, therefore, interpretations) within archival material. After all, disability is there, conflated with disease and the shadow underside of aging, illness, accident, and rehabilitation. It is an uneasy partner serving to hold a mirror up to health. Health looks rather abstract within a more or less disembodied public.

Navigating the archives with these methodological shifts in place offered us important, affective counterbalances and, at times, resistance to dominant narratives of public health that often fell short of explaining the capacity of records and archives to amuse, motivate, inspire, anger, and sadden. These methodological insights serve our emancipatory goal of enhancing the possibility of life for disabled people in future health crises. Understanding how disability has been historically rendered as a kind of void, as not life, or a threat to normal life, present everywhere yet nowhere directly acknowledged in public health archives, reveals patterns that persist in contemporary practice. And to return to our initial animus, namely, the COVID-19 pandemic, it demonstrated precisely this liminal positionality: disabled people were simultaneously hypervisible as “vulnerable populations” at risk yet invisible as people requiring direct, accessible communication and meaningful inclusion in public health decision-making. So thoroughly conceptualized as the unspoken threat lurking behind any health crisis, our lives through disability, with disability, need not be addressed. Our archival analysis documents and challenges the historical production of this pattern where disability is made present as an object to avoid but never addressed as a communicative subject (Goodley, 2018).

Our findings translate into concrete imperatives for public health communication practice. First, the pattern of disability appearing only as “an object of disease but out as a full subject worthy of interaction” must be actively disrupted. Public health institutions must move from communicating about disabled people as problems to communicating with disabled people as full participants. This is difficult, as disability is so well ensconced as that which should be avoided at all costs in every public health encounter. Second, the archival abundance of internal deliberations, contrasted with the paucity of external communication, suggests that institutions often possess knowledge about disability-needs that they fail to communicate publicly or act upon. In other words, considering the ways in which disability is so taken for granted as in need of avoidance or elimination might start to open up the actuality of life in, with, and through disability. Transparency and accountability mechanisms are required to ensure institutional knowledge translates into accessible, inclusive communication. An accountability mechanism might include asking whether disability appears as anything other than a taken-for-granted threat to health within health care education. Third, the conflation of disability with disease that we found throughout the archives continues to shape public health frameworks in ways that harm disabled people - particularly those whose disabilities are unrelated to infectious disease, but who face increased barriers during health crises.

While this paper and other endeavours actively work to ensure these insights reach those who practice public health communication, there is a readily available public health education message, namely, that all involved need to start asking – how does disability figure in research and education other than as a synonym or consequence of disease? This is a difficult question to ask, so our emancipatory commitment is to reveal how thoroughgoing the limited collective imaginary surrounding disability within health contexts is, and to extend this interest into the research process itself. By foregrounding the interpretive work, our difficult choices, and researcher positionalities that shaped our analysis, we model a research practice that acknowledges disability epistemologies. Finally, an acknowledgement of access and affect also became an ethical imperative, soliciting the participation of the reader in the disability archival endeavour.

## Data Availability

The data that support the findings of this study are available as figures embedded in the article body. All figures have alt-text embedded.

## Footnotes

1 Institute of Medicine. (2003). *The Future of the Public’s Health in the* 21st *Century*. Retrieved from https://www.ncbi.nlm.nih.gov/books/NBK221223/. Accessed April 18, 2025.

2 This research project was funded in whole or in part by a Catalyst grant for “Who is speaking to Whom and How? Revisiting Pandemic Communication & Disability” from the University of Toronto’s Institute of Health Emergencies and Pandemics (formerly Institute for Pandemics, 2023-24). Further, the second author acknowledges SSHRC for funding “Reimaging the Dis/Appearances of Disability in the Academy” and acknowledges and thanks the Wellcome Trust for their funding of *Disability Matters* [226705/z/22/z], which has provided time to work on the writing for this project, inclusive of the various ways that the social production and experience of disability influences time in both authors’ lives.
